# *Notes from the Field*: Zika Virus-Associated Neonatal Birth Defects Surveillance — Texas, January 2016–July 2017

**DOI:** 10.15585/mmwr.mm6631a5

**Published:** 2017-08-11

**Authors:** Noemi Borsay Hall, Kelly Broussard, Nicole Evert, Mark Canfield

**Affiliations:** ^1^Epidemic Intelligence Service, CDC; ^2^Texas Department of State Health Services, Austin Texas.

On November 28, 2016, the Texas Department of State Health Services (Texas DSHS) reported its first confirmed case of local mosquitoborne Zika virus transmission in the city of Brownsville, located in south Texas along the U.S.-Mexico border. Zika virus infection during pregnancy has been linked to adverse congenital outcomes including microcephaly, neural tube defects, early brain malformations, structural eye abnormalities, congenital deafness, and limb contractures (*1*). On January 1, 2016, Texas DSHS established enhanced surveillance to identify women with laboratory evidence of possible Zika virus infection during pregnancy and suspected cases of Zika virus–associated birth defects among completed pregnancies.

Relevant epidemiologic information, comprising arboviral disease case investigation findings (including pregnancy status and likely location of exposure) and laboratory test results is collected and reviewed by the Texas DSHS Zoonosis Control Branch as a part of routine arboviral disease surveillance. Each week, the Zoonosis Control Branch shares a line list of pregnant women with laboratory evidence of possible Zika virus infection and their reported pregnancy outcomes with the Texas DSHS Birth Defects Epidemiology and Surveillance Branch. Among possible cases with no reported pregnancy outcome, recent birth certificate data are searched for the reported pregnant woman’s name and birth date to determine whether a live birth has occurred. Birth Defects Epidemiology and Surveillance Branch staff members review neonatal medical records of all babies born to women with possible Zika virus infection during pregnancy to identify all documented birth defects.

During January 1, 2016–July 31, 2017, a total of 219 pregnant women with laboratory evidence of possible recent Zika virus infection were identified in Texas, including 49 (22%) with laboratory-confirmed Zika virus infection (Table). One woman was infected in Texas; all others were exposed outside the United States and its territories. Among the 219 pregnancies, outcomes were recorded for 185 (84%), including 182 live-born infants and three pregnancy losses that occurred at any time during gestation. Among the remaining 34 pregnant women, 20 have an estimated due date which has not yet passed, four have an estimated due date which has passed but no pregnancy outcomes have been reported, and for 10, there was no reported estimated due date. All recorded completed pregnancies were reviewed by the Zoonosis Control and Birth Defects Epidemiology and Surveillance Branches to ascertain Zika virus testing status and to identify any birth defects. Zika virus testing was completed for 80 (43%) of the 185 infants or fetuses, and Zika virus–associated birth defects were documented in 15 (8%) pregnancies (14 live-born infants and one fetal loss), including six (17%) of the 36 infants or fetal losses delivered by women with laboratory-confirmed Zika virus infection. Ten infants or fetuses had microcephaly; five of those with microcephaly had additional birth defects, including holoprosencephaly, hydranencephaly, craniosynostosis, and clubfeet. Zika virus–associated birth defects identified in the remaining three infants included holoprosencephaly, cataracts, and ventral pons hypoplasia.

Zika virus testing was not completed for 105 (57%) infants or fetuses; including three pregnancy losses and 10 live-born infants for whom only a placental or cord blood specimen was tested. In the absence of other evidence, testing of cord blood is insufficient to determine an infant’s infection status (*2*). Placental testing only provides information regarding possible maternal Zika virus infection and cannot confirm or exclude congenital Zika virus infection (*3*). Specimens from 13 infants were unsatisfactory for testing (specimens arrived at an incorrect temperature) or were of insufficient quantity to conduct testing. For the remaining 79 infants, no reason was reported for not conducting Zika virus testing.

The occurrence of travel-related Zika virus infections, combined with the threat of local transmission in Texas, indicates a need for continued surveillance for birth defects associated with Zika virus infection. This analysis found that only 43% of identified infants or fetuses for whom testing was indicated received testing. Efforts to increase the frequency of collecting and testing of specimens from infants born to mothers with laboratory evidence of possible recent Zika virus infection are needed. Physicians caring for newborn infants need to be aware of the Zika testing status of the mother, particularly in geographic locations with high potential for local mosquitoborne transmission. Serum specimens are strongly preferred to placenta or cord blood specimens for infant testing, and should be collected soon after birth (*2*). Neuroimaging before hospital discharge is also recommended for infants born to mothers with evidence of Zika virus infection during pregnancy to detect subtle findings (e.g., calcifications) that indicate congenital Zika infection (*2*). Affected infants should be referred for appropriate clinical and intervention services (*2*).

**TABLE:** Zika virus-associated neonatal birth defects among live-born infants and fetal losses delivered by pregnant women with evidence of Zika virus infection during pregnancy — Birth Defects Epidemiology and Surveillance Branch, Texas Department of State Health Services, January 2016–July 2017

Characteristic	No. (%)		
	Total	Laboratory evidence of possible recent maternal Zika virus infection*	Laboratory-confirmed maternal Zika virus infection^†^
**Pregnant women**	**219 (100)**	170 (78)	49 (22)
Completed pregnancies	**185 (84)**	149 (81)	36 (19)
Live-born infants^§^	**182 (98)**	147 (81)	35 (19)
Pregnancy loss^§^	**3 (2)**	2 (67)	1 (33)
**Zika-associated birth defects** ^§^	**15 (8)**	9 (60)	6 (40)
Microcephaly	**10^¶^ (67)**	6 (60)	4 (40)
Other Zika-associated birth defects	5** (33)	3 (60)	2 (40)
**Infant/Fetus received testing for Zika**	**80^††^ (43)**	57 (71)	23 (29)

* Recent Zika virus infection detected by a positive Zika virus RNA Nucleic Acid Test (NAT) (e.g., reverse transcription-polymerase chain reaction [RT-PCR]) on any maternal, placental, or fetal/infant specimen or detection of recent Zika virus infection or recent unspecified flavivirus infection by serologic tests on a maternal or infant specimen (i.e., either positive or equivocal Zika virus immunoglobulin M [IgM] and Zika virus plaque reduction neutralization test [PRNT] titer ≥10, regardless of dengue virus PRNT value or negative Zika virus IgM, and positive or equivocal dengue virus IgM, and Zika virus PRNT titer ≥10, regardless of dengue virus PRNT titer). Those persons who meet lab-confirmed criteria are not represented among those who have laboratory evidence of possible recent maternal Zika virus infection.

^†^ Zika virus RNA documented by a positive NAT in a maternal, placental, or fetal/infant specimen or detection of recent Zika virus infection by serologic tests on a maternal or infant specimen (i.e., Zika virus IgM was positive or equivocal and Zika virus PRNT titer was ≥10 and dengue virus PRNT was <10).

^§^ Among completed pregnancies, including live-born infants and fetal losses at any time during gestation.

^¶ ^Five of these infants had additional birth defects including holoprosencephaly, hydranencephaly, craniosynostosis, and clubfeet.

** Includes holoprosencephaly, ventriculomegaly, cataracts, choroid plexus cysts, and ventral pons hypoplasia.

^††^Testing not completed for 105 (57%) infants or fetuses, including three pregnancy losses, and 10 live-born infants for whom only a placental or cord blood specimen was tested; 13 specimens could not be tested because the specimens were unsatisfactory, and for the remaining 79 infants, the reason for not testing was not provided.
