# *Erratum*: Vol. 66, No. 31

**DOI:** 10.15585/mmwr.mm6637a9

**Published:** 2017-09-22

In the report “Notes from the Field: Zika Virus-Associated Neonatal Birth Defects Surveillance — Texas, January 2016–July 2017,” on page 835, the final sentence in the third paragraph should have read “Zika virus-associated birth defects identified in the remaining **five** infants included holoprosencephaly, cataracts, and ventral pons hypoplasia.”

